# Recent Advances in the Prevention and Screening of Preeclampsia

**DOI:** 10.3390/jcm12186020

**Published:** 2023-09-17

**Authors:** Balázs Mészáros, Zoltán Kukor, Sándor Valent

**Affiliations:** 1 Department of Obstetrics and Gynecology, Semmelweis University, 1082 Budapest, Hungary; 2 Department of Molecular Biology, Institute of Biochemistry and Molecular Biology, Semmelweis University, 1082 Budapest, Hungary

**Keywords:** preeclampsia, prevention, screening, review, statin, pravastatin, endothelial dysfunction, calcium, vitamin D, Mediterranean-style diet, artificial intelligence, AI, early detection

## Abstract

Throughout the history of medicine, preeclampsia has remained an enigmatic field of obstetrics. In 2023, despite its prevalence and impact, preeclampsia’s exact cause and effective treatment remain elusive; the current options are limited to delivery. The purpose of this review is to summarize the knowledge of the possible novel prophylactic therapies and screening methods for preeclampsia, thereby providing valuable insights for healthcare professionals and researchers. Aspirin and LMWH have already been widely used; meanwhile, calcium, vitamin D, and pravastatin show promise, and endothelin receptor antagonists are being explored. Stress reduction, dietary changes, and lifestyle modifications are also being investigated. Another interesting and fast-growing area is AI- and software-based screening methods. It is also key to find novel biomarkers, which, in some cases, are not only able to predict the development of the disease, but some of them hold promise to be a potential therapeutic target. We conclude that, while a definitive cure for preeclampsia may not be eligible in the near future, it is likely that the assessment and enhancement of preventive methods will lead to the prevention of many cases. However, it is also important to highlight that more additional research is needed in the future to clarify the exact pathophysiology of preeclampsia and to thus identify potential therapeutic targets for more improved treatment methods.

## 1. Introduction

### 1.1. The Epidemiology and the Definition of Preeclampsia

Preeclampsia, a pregnancy-specific condition, affects 2–8% of pregnancies globally and stands as a major contributor to both maternal and fetal illness and death [1,2,3]. As per the International Society for the Study of Hypertension in Pregnancy (ISSHP), preeclampsia is defined as the emergence of hypertension after the 20th week of pregnancy coupled with proteinuria and/or maternal complications, like acute kidney injury (AKI), liver issues, neurological problems, thrombocytopenia, or hemolysis, as well as fetal growth restriction [4].

### 1.2. Theories and Hypotheses on the Pathophysiology of Preeclampsia 

While the exact pathophysiology of the disease is not completely known as of August of 2023, most researchers agree that it is a multifactorial disease which has a genetic and environmental background as well [5,6,7]. Additionally, over the past decade, substantial progress has been made in understanding the pathophysiology of preeclampsia [8].

Based on the prevailing theories, preeclampsia is considered a clinical syndrome that initiates in the early stages of pregnancy, marked by abnormal trophoblast invasion and the remodeling of the uterine spiral arteries. These factors result in abnormal placentation, leading to placental ischemia and the release of antiangiogenic factors. These antiangiogenic factors include soluble fms-like tyrosine kinase-1 (sFlt-1) and soluble endoglin (sEng). Elevated levels of sFlt-1 and sEng lead to vasoconstriction and endothelial dysfunction (which, according to many theories, is not only a consequence of preeclampsia but could also possibly be preexistent and thus be a cause of PE), negatively affecting both the maternal and fetal organs as well as their blood perfusion [9,10,11]. This theory is supported by several studies conducted on animals in which elevated levels of antiangiogenic factors (sFlt-1 and sEng) induced symptoms with characteristics that resembled preeclampsia in the animals. These symptoms included hypertension, proteinuria, the low birth weight of the fetus, and renal and hepatic dysfunctions [12,13]. Not only were antiangiogenic factors’ levels raised, but researchers also found that angiogenic factors’ levels (e.g., VEGF and PlGF) were lowered [14,15,16].

In addition to the already presented endothelial causes, several immunological factors can also play a role in the development of the disease. During uncomplicated pregnancies, the ratio of T helper cells shifts towards the anti-inflammatory Th2 phenotype [17,18]. Studies have emerged that seem to confirm that, in mothers struggling with preeclampsia, this balance shifts towards Th1 cells. Th1 cells are proinflammatory cells, increasing the levels of interleukin-12 and -18 (IL-12 and IL-18) while reducing IL-10 levels. Furthermore, abnormal functioning of the complement system has been detected in preeclampsia patients, which may provide a further explanation for the continued increase in sFlt-1 levels [1,19]. Many pieces of research also claim that oxidative stress has a major role in both placental and endothelial dysfunction, which subsequently lead to preeclampsia [20,21].

Also, in recent years, studies have surfaced that have distinguished novel preeclampsia phenotypes, which could be a huge step toward the personalized therapy of preeclampsia-affected mothers [12,22].

There are also predisposing factors for the development of preeclampsia, and it is essential to highlight chronic hypertension, obesity, and pregestational diabetes mellitus, the prevalence of which is increasing in most developed countries, leading to a growth in preeclampsia cases. Additionally, it has been described that, in certain racial groups, the occurrence of preeclampsia is higher than that in the general population. A low socioeconomic status, as with most diseases, also increases the risk of its development [6,23]. 

As of the present moment, the only definitive treatment for preeclampsia is delivering both the newborn and the placenta [1,24,25]. However, there are a number of treatments that have been used in the clinical practice for a long while to prevent the disease: aspirin has shown efficacy in reducing the risk of preeclampsia, especially among high-risk cases, and LMWH (low-molecular-weight heparin) is considered an effective treatment drug, as it potentially improves the placental blood flow and reduces coagulation-related risks [26,27,28,29]. 

### 1.3. The Purpose of This Current Review

Additionally, several innovative approaches exist that seek to either prevent the onset of the condition or to provide treatment for it. This current review seeks to summarize our current understanding of these novel screening and prophylactic methods. 

## 2. Aspirin and LMWH in Preeclampsia Prevention

To start the topic of preeclampsia prevention, it is important to talk about the most widespread method around the world: low-dose aspirin prophylaxis. The mechanism behind this preventive method lies in aspirin’s ability to inhibit platelet aggregation and to promote vasodilation, ultimately improving the blood flow to the placenta [30,31].

Several studies and clinical trials have explored aspirin’s effectiveness in reducing the incidence of preeclampsia. Notably, the ASPRE (Aspirin for Evidence-Based Preeclampsia Prevention) trial demonstrated that low-dose aspirin initiated during the first trimester of pregnancy could lower the risk of preeclampsia in women at high risk for the condition [32]. Other pieces of research have also indicated that aspirin may have additional benefits not only in preeclampsia, such as reducing the risk of preterm birth and intrauterine growth restriction [33,34]. 

It is also important to study the doses and the timing of aspirin prophylaxis: the recent meta-analysis of Van Doorn et al. found that 81 mg of pravastatin prophylaxis is the most beneficial dose; the meta-analysis was pooled from randomized trials from 1985 to 2019, enrolling cumulatively more than 30,000 patients [35]. If we want to compare the aspirin guidelines for preeclampsia, we see that the dosage in the guidelines ranges between 75 and 150 mg/day, and in the field of timing, the most recommend time to start the therapy is before the 20th gestational week. To highlight two guidelines, the NICE recommends the start of the preventive usage of aspirin in the 12th gestational week with a 75–150 mg daily dosage, while the WHO recommends 75 mg before the 20th gestational week [36].

Clinical trials and studies have provided insights into the potential benefits of LMWH prophylaxis in preeclampsia: According to a 2016 meta-analysis, the addition of LMWH to aspirin therapy could further reduce the odds of preeclampsia in women with a history of preeclampsia [37]. Long et al. also reported in 2023 that low-dose aspirin therapy with the addition of LMWH prophylaxis could potentially delay the onset of severe preeclampsia [38].

However, it is also important to highlight that, even though aspirin and heparin are widely used in preeclampsia and their efficacy was proven through meta-analyses as shown above, there is still no full consensus on the dosage and the start time of aspirin and heparin therapy or on the possible replacement of aspirin with LMWH in specific cases—these facts emphasize further the need to discover novel treatments and prophylaxis methods for preeclampsia [36,39,40,41].

## 3. Calcium Supplements, Vitamin Supplements, and Other Dietary Interventions in Preeclampsia Prevention

As calcium plays a vital role in the function of the blood vessels, helping to regulate the vascular tone and to maintain a healthy blood pressure [42,43,44], and as it also plays a role in placental development and function [45,46], it is logical why calcium has been studied in the prevention and treatment of preeclampsia. A recent meta-analysis that included more than 20,000 patients concluded that calcium supplementation is effective for preeclampsia prevention in women with a low calcium intake [47]. Patrelli et al. published a meta-analysis that also concluded that the increased intake of calcium decreases the chances of the development of preeclampsia [48]. However, though numerous studies indicate the benefits of calcium supplementation in preeclampsia prevention, there remains a lack of consensus regarding the appropriate dosage. Woo Kinshella et al., in their recent meta-analysis, examined randomized controlled trials investigating calcium supplementation for the prevention of preeclampsia. Their findings indicated that there were no significant distinctions between patients receiving high dosages (≥1 g/day) of calcium and those receiving low dosages (<1 g/day) [38].

Not only calcium supplementation but also vitamin supplementation, especially the supplementation of vitamin D, was researched with respect to the prevention of preeclampsia [49,50]. Nema et al. found that vitamin D supplementation enhances angiogenic factors in preeclampsia: it raises the levels of VEGF (vascular endothelial growth factor) and PlGF [51]. According to the meta-analysis of Khaing et al. [52], calcium supplementation and vitamin D may be used for the prevention of preeclampsia, but they also suggest that further research is needed. 

However, it is also important to note that excessive levels of vitamin D and elevated calcium levels are a danger of supplementation and may lead to the formation of calcium stones in the kidney, nausea, digestive issues, and soft-tissue calcification [53,54].

Additionally, vitamin D, vitamin C, and vitamin E supplementation have also been studied with respect to the prevention of preeclampsia. Oxidative stress significantly contributes to the progression of preeclampsia. Vitamins C and E are antioxidants, which is why their protective role in preeclampsia has been investigated for a long time [55,56]. Vitamin C prevents the auto-oxidation of tetrahydrobiopterin, a cofactor of NO synthase [57,58]. Vitamin C can thus indirectly increase the insufficient nitric oxide level in preeclampsia. However, the meta-analysis of Polyzos et al. highly questions the usage of combined vitamin C and E supplementation, as it suggests that such antioxidants do not reduce the risk of developing preeclampsia; they found no significant differences between their control and placebo groups [59]. Three years later, Busaran et al. also found no differences between their control and placebo groups regarding the development of preeclampsia [60]. Contrary to these results, a recent large retrospective clinical trial found that vitamin E and A deficiencies are both risk factors for preeclampsia [61]. 

A recent Cochrane database systematic review also reported the positive role of fish oil (omega-3 fatty acids) intake during pregnancy, lowering the risks of preterm birth [62]. However, its positive role has not been proven in preeclampsia yet [63].

In recent years, a Mediterranean-style diet—a diet that emphasizes the consumption of fruits, vegetables, nuts, and seeds as well as the daily intake of olive oil and whole grains—has gained more and more popularity in the primary and secondary prevention of cardiovascular diseases, and studies indicate that it could also be beneficial in obstetrics prevention [64,65,66,67]. The Boston Birth Cohort study reported the beneficial impact of a Mediterranean-style diet in reducing the odds of preeclampsia. This study involved 8507 women, and positive effects were observed across all the racial groups examined. Notably, the findings suggest that even black women, who face a higher risk of preeclampsia in the United States, can derive benefits from adopting a Mediterranean-style diet during their pregnancies [68].

In conclusion, a low calcium intake and low vitamin D levels appear to enlarge the risk of preeclampsia development, but other vitamins’ role in preeclampsia development should be studied further, as there is no concluding evidence that they are useful in lowering the risk of preeclampsia development. While the intake of omega-3 fatty acids does not seem to reduce the risk of preeclampsia, a Mediterranean-style diet seems to be a beneficial dietary intervention during pregnancy.

## 4. Endothelin Receptor Antagonists

As endothelial dysfunction plays a pivotal role in preeclampsia’s pathophysiology, it could be key to improving one’s endothelial function to lower the risk of the development of preeclampsia and/or to treat the disease [69,70]. Larsen et al. have observed the emergence of autoantibodies targeting Angiotensin II receptor 1 and the endothelin receptor in women experiencing preeclampsia [71]. As a result, endothelin receptor antagonists have been tested to prevent preeclampsia [72,73]. 

A notable contribution in this direction comes from the work of Hitzerd et al., who published the first instance of directly transferring endothelin receptor antagonists to the human placenta. This significant step indicates the feasibility of targeting placental endothelin receptors as a therapeutic strategy for preeclampsia [74].

Although more research is needed, the prospect of utilizing endothelin receptor antagonists to address endothelial dysfunction in the context of preeclampsia holds great promise. By targeting the endothelin receptor system, these antagonists have the potential to mitigate the underlying vascular and placental abnormalities that contribute to preeclampsia development. While the path ahead requires rigorous investigation and clinical validation, endothelin receptor antagonists represent a compelling avenue for advancing preeclampsia treatment strategies in the future.

## 5. The Usage of Statins in Preeclampsia

The rate-limiting step of cholesterol synthesis is the conversion of HMG-CoA to mevalonate, an action facilitated by HMG-CoA reductase. Statins function as competitive inhibitors of this enzyme, leading to an effective reduction in cholesterol levels within the bloodstream—in this way, they are widespread in cardiology in the primary and secondary prevention of acute myocardial infarction (AMI) [75]. However, in recent years, professionals have also been studying them in the field of obstetrics and have been gaining promising results with them in the treatment and the prevention of preeclampsia. Many studies indicate that statins also raise the levels of PlGF (placental growth factor); in this way, they reduce the levels of sFlt-1, thus helping to fight against the antiangiogenic factors that cause preeclampsia [76,77]. There are also studies which suggest that statins enhance microsomal nitrogen monoxide synthesis, and that is the main reason that they have a positive effect on microsomal circulation [78,79,80]; also, there are studies which have shown that statins raise VEGF levels, thus helping the vascularization of the placenta [19,81].

As it is known, there are lipophilic and hydrophilic statins; hydrophilic statins (rosuvastatin and pravastatin) have more favorable pharmacokinetics and appear to be less teratogenic than lipophilic ones [82,83]. The first pieces of evidence that were found of the positive effects of pravastatin in preeclampsia were concluded in animal models: Saad et al. [84] showed pravastatin’s positive effect in pregnant CD1 mouse models, while Carver et al. [85] did so with a murine preeclampsia model. The initial human studies consisted of case reports. In 2014, Lefkou et al. reported the case of a preeclamptic mother who received the triple therapy of pravastatin, aspirin, and enoxaparin. The mother delivered a healthy infant, and both of them remained free of adverse effects [86]. In another study, Saito et al. presented two cases where women with a medical history of APS (antiphospholipid syndrome) and systemic lupus erythematosus (SLE), along with a history of preeclamptic and unsuccessful pregnancies, received pravastatin therapy. These women remained symptom-free and experienced no adverse effects during their pregnancies that were examined in the study [87].

Kupferminc et al. conducted a retrospective cohort study involving 32 women who had experienced severe placenta-mediated complications in previous pregnancies, who served as the control group. These women were administered a pravastatin treatment beginning in the 12th week of gestation. Among the control pregnancies, 17 instances of severe preeclampsia were recorded. In contrast, among the pregnancies where pravastatin was used, only two cases of preeclampsia were identified, and the symptoms observed in these instances were of a mild nature [88].

The INOVASIA study also reported favorable outcomes in the secondary prevention of preeclampsia with the usage of pravastatin: the rates of preterm delivery and neonatal morbidity were significantly lower, while the birth weight and Apgar scores were significantly higher in the group in which mothers received pravastatin instead of a placebo [89,90,91].

Costantine et al. also carried out two clinical trials with the usage of pravastatin in preeclampsia: women who were at high risk of preeclampsia received pravastatin therapy before the 20th gestational week; both of the studies confirmed the overall safety and favorable outcomes of pravastatin [92,93].

Even though these reports suggest pravastatin therapy is useful before the 20th gestational week, a study carried out by Döbert et al., where 1120 women were assigned and received either pravastatin or a placebo after the 35th gestational week, suggests that, this late in the pregnancy, pravastatin does not reduce the incidence of preeclampsia [94].

In a meta-analysis that was carried out by our research group, we also concluded that pravastatin use in high-risk populations prior to the 20th gestational week decreases the risk of preeclampsia and appears to decrease the risk of preterm birth, IUGR (intrauterine growth restriction), and NICU (neonatal intensive care unit) admissions among neonates. However, as there were not sufficient data [95], to conclude the meta-analysis, after the 20th gestational week, further clinical studies and meta-analyses need to be carried out not only in terms of prevention but also in the treatment efficacy of pravastatin for preeclampsia.

In addition, in a long-term neurodevelopmental follow-up study by Costantine et al., no differences were observed between the children of mothers who received pravastatin during pregnancy and those whose mothers did not receive the medication [96].

While the FDA (US Food and Drug Administration) continues to advise against the regular application of statins throughout pregnancy, a notable shift occurred in July 2021. The FDA requested the elimination of the longstanding restriction on employing statins in women who are pregnant or planning to become pregnant. This modification holds particular significance for healthcare practitioners tending to women of a reproductive age who have familial hypercholesterolemia or a background of atherosclerotic cardiovascular conditions [97].

## 6. New Approaches to the Pregnancy Lifestyle

Even though the relationship between larger BMIs and a higher incidence of preeclampsia as well as a more severe course of the disease has been known for decades [98,99,100], a healthy lifestyle and lifestyle changes during pregnancy have been in the focus more than probably ever in human history [101,102,103,104]. It is important to highlight a meta-analysis from 2018 that found, based on more than 100 clinical trials and more than 250,000 patients’ data, that exercise-only interventions lower the risk of many pregnancy-related adverse outcomes, including preeclampsia (*n* = 3322, OR of 0.59, 95% CI of 0.37 to 0.9), compared to the data of patients who performed no exercise during their respective pregnancies [105]. A 2023 meta-analysis carried out by Liu et al. also found that exercise during pregnancy lowers the risk of preeclampsia [106]. As per the 2021 meta-analysis conducted by Magro-Malosso et al., engaging in aerobic exercise sessions lasting around 30 to 60 min occurring two to seven times per week throughout pregnancy has shown a link to a notable decrease in the overall risk of gestational hypertensive disorders. This finding highlights the potential benefits of moderate physical activity in lowering the odds of such conditions when compared to a more sedentary lifestyle [107].

When we talk about lifestyle, it is also important to mention not only sports but working hours as well: Cai et al. found that women who work rotating shifts, fixed night shifts, or longer hours during their pregnancies have an increased risk of adverse pregnancy outcomes, including preeclampsia [108]. Another meta-analysis from the same work group also found that demanding physical work during pregnancy raises the incidence of adverse pregnancy outcomes: women who face the occupational risks of heavy lifting, prolonged standing, prolonged walking, prolonged bending, and a heavy physical workload are more likely to face preeclampsia during their pregnancies compared to those that are not exposed to the above-mentioned factors [109]. Reducing stress can also be beneficial in preeclampsia prevention [110].

In essence, the view around lifestyle interventions during pregnancy has evolved significantly. While the association between BMI and preeclampsia remains well established, recent research emphasizes the pivotal role of adopting a holistic approach to lifestyle modifications. Exercise stands as a key factor in preventing adverse pregnancy outcomes, including preeclampsia. Furthermore, considerations beyond physical activity, such as working hours and stress management, bring additional layers of complexity to the quest for effective preeclampsia prevention. As the field continues to advance, integrating these multifaceted lifestyle interventions into prenatal care could usher in a new era of maternal well-being and healthier pregnancies.

## 7. Novel Markers in Preeclampsia

Recent advancements in preeclampsia research have led to the identification of novel markers that not only are helpful in detecting the disease earlier but also hold promise in enhancing our understanding of it. These emerging markers include a range of biological molecules, specific proteins, micro-RNAs, and metabolites [111].

Elevated levels of visfatin, which is also known as nicotinamide phosphoribosyltransferase (NAMPT), in preeclamptic pregnancies compared to healthy and normotensive pregnancies have been investigated as a potential biomarker due to its role in inflammation and metabolism [112].

Uric acid has also gained global attention in the guidelines for hypertensive disorders of pregnancy (HDP) and preeclampsia as an important factor for assessing risk [113,114]. Recent studies have highlighted the usefulness of the uric-acid-to-creatinine ratio as a new way to predict preeclampsia. This method is an improvement, as it goes beyond only looking at uric acid levels in the blood. Instead, it considers the functions of the kidneys. This holds significance, as it enables the identification of individuals who could exhibit elevated uric acid levels owing to both renal impairment and heightened oxidative stress [115].

Another possibly important yet understudied marker is allopurinol—only one trial has investigated the impacts of allopurinol in high-risk pregnancies, yielding contradictory outcomes, partly attributed to the study design [116].

## 8. Micro-RNAs and Preeclampsia

Pregnancy-specific micro-RNAs (miRs) are produced during pregnancy. These miRs are primarily expressed by the trophoblast and the placenta and are packaged in exosomes; they create a connection between the mother and the fetus/placenta. The C14MC, C19MC, and miR-371–3 clusters can be considered to be pregnancy-specific; their measurable concentration in the blood increases strongly during pregnancy. C14MC is highly expressed in the first trimester and decreases in the third trimester, whereas the expression of C19MC and miR-371-3 rises towards the end of pregnancy [117]. C14MC is located on the 14q chromosome. C19MC and miR-371–373 are located on the 19q chromosome. C19MC is primate-specific [117]. It is conceivable that changes in the expression of C19MC contribute to the fact that preeclampsia is a disease characteristic only of primates. M19MC miRs are produced in the largest amount during pregnancy and are intensively studied. The miR-371-3 cluster consists mainly of three miRNAs. The role of miR-371-3 is currently less well known [118,119], and we are not aware of any studies pertaining to preeclampsia.

More than one hundred micro-RNAs whose expression changes in preeclampsia have already been identified. The small number of samples of the tests, the different measurement techniques, and their presentation do not yet allow their practical applicability. Primarily, they can play a role in clarifying the prediction of preeclampsia, but there are experiments that could establish a possible therapeutic application [118]. The marked difference in the change in C14MC and C19MC expression in preeclampsia is noteworthy. In preeclamptic placentas, C14MC is predominantly downregulated, while C19MC miRs are upregulated [120].

A predictive value was found between circulating miRNAs circulating before 20 weeks of pregnancy and preeclampsia [121]. On https://clinicaltrials.gov/search?cond=Preeclampsia&intr=miRNA (accessed on 7 September 2023), there is no trial (September 2023) investigating the therapeutic use of miRs. Realistically, miRs could be used to predict preeclampsia. For this purpose, maternal serum or plasma can be used noninvasively. Table 1 contains the changes in the serum miR concentration collected before the 20th week of pregnancy. We selected those miRs in the list where the methodology clearly describes that the sample was collected before the 20th week.

There is a high level of the circulating fractions of C19MC micro-RNAs in the serum and plasma in the first trimester in preeclamptic women. Only for miR-525-5p, low levels of plasma expression were noted in the first trimester. The miRNAs belonging to cluster C19MC could be promising biomarkers of preeclampsia development [129]. Based on the data in the table, it can be considered to be clear that concentration changes can cause the inhibition of the trophoblast invasion and migration characteristics of preeclampsia and can have an antiangiogenic effect.

## 9. Early Detection and Monitoring with the Help of Rapidly Advancing Softwares and Artificial Intelligence (AI)

Finally, we would like to introduce the prominently improving softwares and AI (artificial intelligence) methods that are most helpful in the prediction of the risk of preeclampsia, this way allowing physicians closer surveillance and to intervene earlier [130].

In these softwares, physicians can type in the data of the mothers (e.g., age, height, weight, race, family history, smoking, preliminary type I or II diabetes, preexisting chronic hypertension, SLE, and APS) and also the biophysical measurements of the fetus (e.g., mean arterial pressure and mean uterine artery PI); some softwares even count with the above-mentioned preeclampsia biomarkers, such as serum PlGF and serum PAPP-A (pregnancy-associated plasma protein A). If all the available data are typed in, the program gives the approximate risk to the doctor for the development of preeclampsia later during the pregnancy. Some of these programs are also available in the form of a mobile phone application, this way being more accessible to professionals [131,132,133]. Grünebaum et al. also claim that ChatGPT could have a massive educational role in obstetrics and gynecology as well, both on the patients’ and professionals’ side [134].

**Figure 1:**
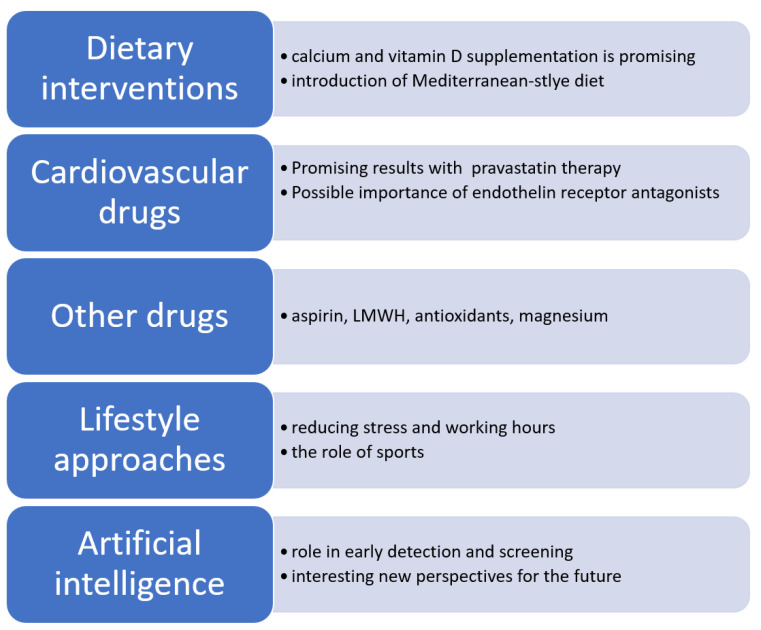
The summarization of preeclampsia preventions and therapies presented in this review.

A summarization of the possible preeclampsia preventions and therapies is presented in Figure 1.

## 10. Conclusions

The development of novel preventive strategies is crucial, given the severe consequences of preeclampsia. 

Several promising approaches have been explored, including the supplementation of calcium and vitamin D, the potential benefits of a Mediterranean-style diet, and the evaluation of endothelin receptor antagonists. These interventions show potential in reducing the risk of preeclampsia and improving maternal outcomes. As the results are controversial with other vitamins, it would be desirable if larger clinical trials and meta-analyses were carried out by professionals in the near future.

Pravastatin has also shown promise in early pregnancy for lowering the risk of preeclampsia. However, more clinical trials are needed to fully validate its safety and effectiveness, particularly in late pregnancy.

Lifestyle factors, such as regular exercise, stress reduction, and healthy and stress-free working hours, play a critical role in preventing preeclampsia and promoting overall maternal well-being.

Novel preeclampsia markers, including specific proteins, micro-RNAs, and metabolites, hold potential for both diagnostic and therapeutic applications. Notably, altered micro-RNA profiles in placental tissues and the maternal circulation of preeclamptic pregnancies suggest their value as both biomarkers and therapeutic targets. Uric acid’s significance in assessing the risk of a hypertensive disorder is emphasized, with the uric-acid-to-creatinine ratio emerging as a more comprehensive predictor due to its consideration of kidney function.

The integration of artificial intelligence (AI) and software-based screening methods is a significant advancement of the past few years in the early detection of preeclampsia. These tools enable more vigilant surveillance and timely interventions for physicians. Their above-mentioned characteristics likely will make them more widespread in the future clinical practice.

This article demonstrates that, while the only definitive treatment for preeclampsia remains the delivery of the neonate and placenta, significant progress has been made, particularly in preventing and screening for preeclampsia. Our review indicates that a substantial portion of patients can derive advantages from these measures if more information is gained and these measures are used with professionalism and care. It is also likely that, while preeclampsia cannot be cured in the near future, with the evaluation and improvement of the preventive methods, a lot of cases can be prevented.

Finally, it is also important to highlight the role of preclinical research. As our understanding of the underlying mechanisms of preeclampsia improves, it may lead to the discovery of new therapeutic targets and approaches. As our understanding will hopefully evolve, in the near future, healthcare professionals and researchers must work collaboratively to enhance the management of this complex condition, ultimately reducing its impact on maternal and fetal health.

## Figures and Tables

**Table 1:** Changes in plasma levels of maternal miRs before the 20th week of pregnancy. ↓ level decrease; ↑ level increase; JAR cell line—human choriocarcinoma cell line. The miRs were selected based on the publications of Qin et al. (2022) [121] and Zhafir Asyura et al. (2023) [119].

miR	Cluster	Change	Potential Effect of Changed miR in Preeclampsia
miR-517-5p	C19MC	↑	inhibits the proliferation and invasion of JAR cell line [122]
miR-942	-	↓	inhibition of trophoblast invasion and angiogenesis [123]
miR-517a	C19MC	↑	decreased trophoblast invasion and antiangiogenic effect [123]
miR-517c	C19MC	↑	decreased trophoblast invasion and antiangiogenic effect [123]
miR-518b	C19MC	↑	inhibits trophoblast migration and angiogenesis [124]
miR-516b-5p	C19MC	↑	N.D.
miR-520a-5p	C19MC	↑	N.D.
miR-520h	C19MC	↑	N.D.
miR-525-5p	C19MC	↑ [125] ↓ [126]	overexpression mediates the invasion of trophoblast cells [123]
miR-125b	-	↑	inhibits cytotrophoblast invasion and impairs endothelial cell function [127]
miR-126-3p	-	↓	inhibits trophoblast proliferation and promotes trophoblast apoptosis [128]

## Data Availability

All data used are available upon request. Please contact the corresponding author, Zoltán Kukor, for access.

## Abbreviations

AI—artificial intelligence, AKI—acute kidney injury, APS—antiphospholipid syndrome, BMI—body mass index, ERA—endothelin receptor antagonist, FDA—US Food and Drug Administration, IL-10—interleukin-10, IL-12—interleukin-12, IL-18—interleukin-18, ISSHP—International Society for the Study of Hypertension in Pregnancy, IUGR—intrauterine growth restriction, LMWH—low-molecular-weight heparin, NAMPT—nicotinamide phosphoribosyltransferase, NICU—Neonatal Intensive Care Unit, PAPP-A—pregnancy-associated plasma protein A, PI—pulsatility index, PlGF—placental growth factor, sEng—soluble endoglin, sFlt-1—soluble fms-like tyrosine kinase-1, SLE—systemic lupus erythematosus, and VEGF—vascular endothelial growth factor.
